# Draft Genome Sequence of the Alkaliphilic and Xylanolytic *Paenibacillus* sp. Strain JCM 10914, Isolated from the Gut of a Soil-Feeding Termite

**DOI:** 10.1128/genomeA.01144-13

**Published:** 2014-01-23

**Authors:** Moriya Ohkuma, Masahiro Yuki, Kenshiro Oshima, Wataru Suda, Yumi Oshida, Keiko Kitamura, Toshiya Iida, Masahira Hattori

**Affiliations:** a Japan Collection of Microorganisms/Microbe Division, RIKEN BioResource Center, Tsukuba, Japan; b Biomass Research Platform Team, RIKEN Biomass Engineering Program, Tsukuba, Japan; c Center for Omics and Bioinformatics, Graduate School of Frontier Sciences, the University of Tokyo, Kashiwa, Japan

## Abstract

*Panibacillus* sp. strain JCM 10914 is a xylanolytic alkaliphile isolated from the gut of a soil-feeding termite. Its draft genome sequence revealed various genes for hydrolytic enzymes and will facilitate studies on adaptation to the highly alkaline gut environment and its role in digesting soil organic matter in the gut.

## GENOME ANNOUNCEMENT

*Paenibacillus* sp. strain JCM 10914 (originally described as strain SM-XY60 and available from the Japan Collection of Microorganisms [JCM] [http://www.jcm.riken.jp]) was isolated from the gut of a soil-feeding termite, *Sinocapritermes mushae* (Isoptera; Termitidae) (1). Gut portions of soil-feeding termites generally show extreme alkalinity, containing large numbers of potassium ions (2, 3). Gut alkalinity is effective for solubilization of soil organic matter and facilitates its utilization (4). Bacterial species closely related to this strain have been detected in the alkaline portion of the gut in several soil-feeding termites (5).

The optimal growth of this strain is around pH 9 in medium containing 1% K_2_CO_3_. Although alkaliphiles have usually been isolated in alkaline media containing Na_2_CO_3_, this strain prefers potassium ions to sodium ions; indeed, growth is inhibited in the alkaline medium of 1% Na_2_CO_3_ or in the presence of 5% NaCl at a neutral pH (1). This strain exhibits strong xylanolytic ability only when the cells are grown under alkaline conditions, and the produced xylanases have substantial activity and stability at high pH (1). This strain also shows weaker but significant cellulolytic ability. The potassium-preferred alkaliphilic nature and the xylanase produced at high pH are probably a consequence of the strain’s adaptation to the extremely alkaline gut environment.

The whole-genome sequencing of *Paenibacillus* sp. strain JCM 10914 was carried out using the Ion Torrent PGM System with 200-bp read chemistry (Life Technologies). Using Newbler version 2.8 (Roche), we removed low-quality and short reads and assembled the remaining 620,497 reads into 95 contigs with an *N*_50_ length of 153,857 bp and the longest length of 706,562 bp. This assembly resulted in a draft genome sequence of 6,112,248 bp with 22.5× redundancy and a G+C content of 48.6%. A total of 6,080 protein-encoding genes and 69 RNA-encoding sequences were detected after manual inspection of the annotations using the RAST server (6).

A single full-length (1,545-bp) 16S rRNA gene sequence was obtained from this annotation. Phylogenetic analysis based on the 16S rRNA gene sequence revealed the closest relatedness of strain JCM 10914 to type strains of *Paenibacillus glucanolyticus* and *Paenibacillus lautus*, sharing 97.0% and 96.9% identity, respectively. Preliminary analyses revealed the presence of genes encoding xylanases homologous to those in the glycoside hydrolase (GH) 8, 10, and 43 families in the CAZy database (7), genes encoding α- and β-xylosidases of GH31 and 43, respectively, genes encoding cellulases of GH5 and 9, and genes encoding β-glucosidase of GH3. These xylanolytic and cellulolytic enzymes have potentials for biotechnological applications, as do many enzymes of alkaliphiles (8).

### Nucleotide sequence accession numbers.

The genome sequence of *Paenibacillus* sp. JCM 10914 has been deposited in DDBJ under the accession numbers BAUO01000001 to BAUO01000095.
